# Transitioning from electrodialysis to reverse electrodialysis stack design for energy generation from high concentration salinity gradients

**DOI:** 10.1016/j.enconman.2021.114493

**Published:** 2021-09-15

**Authors:** A.M. Hulme, C.J. Davey, S. Tyrrel, M. Pidou, E.J. McAdam

**Affiliations:** Cranfield Water Science Institute, Cranfield University, Bedfordshire MK43 0AL, UK

**Keywords:** Reverse electrodialysis, Electrodialysis, Intermembrane distance, Ion exchange membranes, Concentrated brines, Salinity gradient energy

## Abstract

In this study, stack design for high concentration gradient reverse electrodialysis operating in recycle is addressed. High concentration gradients introduce complex transport phenomena, which are exacerbated when recycling feeds; a strategy employed to improve system level energy efficiency. This unique challenge indicates that membrane properties and spacer thickness requirements may differ considerably from reverse electrodialysis for lower concentration gradients (e.g. seawater/river water), drawing closer parallels to electrodialysis stack design. Consequently, commercially available electrodialysis and reverse electrodialysis stack design was first compared for power generation from high concentration gradients. Higher gross power densities were identified for the reverse electrodialysis stack, due to the use of thinner membranes characterised by a higher permselectivity, which improved current. However, energy efficiency of the electrodialysis stack was twice that recorded for the reverse electrodialysis stack at low current densities, which was attributed to: (i) an increased residence time provided by the larger intermembrane distance, and (ii) reduced exergy losses of the electrodialysis membranes, which provided comparatively lower water permeance. Further in-depth investigation into membrane properties and spacer thickness identified that membranes characterised by an intermediate water permeability and ohmic resistance provided the highest power density and energy efficiency (Neosepta ACS/CMS), while wider intermembrane distances up to 0.3 mm improved energy efficiency. This study confirms that reverse electrodialysis stacks for high concentration gradients in recycle therefore demand design more comparable to electrodialysis stacks to drive energy efficiency, but when selecting membrane properties, the trade-off with permselectivity must also be considered to ensure economic viability.

## Highlights

• Reverse electrodialysis stack design studied for large salinity gradients in recycle.
• Energy efficiency of a Reverse electrodialysis and Electrodialysis stack compared.
• Thicker electrodialysis membranes exhibited lower water permeance.
• Membrane properties and intermembrane distance critical for high concentrations.

## 1 Introduction

Electrodialysis (ED) is a commercially mature technology, with applications in multiple industries ranging from the food industry to wastewater treatment [1]. Through revisiting stack design, ED has been demonstrated to be economically [2] and energetically [3] competitive to reverse osmosis for the desalination of brackish waters. In an ED stack, anion and cation ion exchange membranes are alternately arranged between two electrodes to form concentrate and dilute compartments, with spacers and gaskets separating the membranes. The controlled movement of ions across the ion exchange membranes (IEMs) is driven by an applied electrical current to produce desalinated water [4]. In reverse electrodialysis (RED), the opposite process to ED is employed, where ionic transport across alternately stacked IEMs is driven by a concentration gradient, to liberate the Gibbs free energy of mixing between solutions of different salinities. A redox couple circulating across the electrodes converts the ionic flow to an electric current [5].

Whilst sharing mechanistically comparable separation principles, the modules used for desalination by ED and those used for power production by RED exhibit several key differences. An intermembrane distance of 0.3 mm to 2 mm is typically used in ED modules [1], as the increased volume of salt solution enables a higher potential to be applied [6]. In contrast to ED, compartment widths in the region of 0.1 mm to 0.3 mm have typically been adopted for RED stacks [7]. Vermaas et al. [8] demonstrated that reducing resistance through decreasing the intermembrane distance doubles power density using sea/river water feeds. Another difference is in the ion exchange membranes used for ED and RED. In ED, low water permeability is critical to reduce energy consumption and minimise water transport which occurs by: (i) osmosis, facilitated by the concentration difference across the membrane; and (ii) electro-osmosis, in which ionic transport across the membrane facilitates the co-transport of associated water molecules [9]. Conversely, in RED, electro-osmosis occurs in the opposite direction to osmosis and thus acts to reduce net water transport [10]. A theoretical analysis of ion exchange membranes established that low resistance membranes characterised by high permselectivity should be prioritised for RED applications using artificial sea and river water [11]. This was confirmed by the experimental evaluation of tailor-made [12] and commercially available membranes [13]. Consequently, RED stack design has migrated away from conventional ED stack design for feed waters of equivalent salinity by introducing membranes which promote higher permselectivity and reduced resistance at the cost of water transport to improve power density [5]. However, this research utilised seawater and river water to develop the concentration gradient driving force, with power densities up to 0.93 W m^−2^ obtained using these feeds [14]. Higher salinity differences (e.g. desalination reject brine) [15], increase the system electrochemical potential (Nernst potential), with power densities up to 6.7 W m^−2^ reported at elevated temperatures [16]. However, whilst the effects of water transport are less evident in RED compared with ED when seawater/river water is used, such phenomena can conceivably pose a significant resistance to ionic transport when higher salinity gradients are employed, increasing the driving force for osmosis [17].

For energy storage and thermal-to-electric conversion systems, closed-loop RED is proposed, in which mixed solutions exiting the RED stack subsequently pass through a separation stage which restores the concentration gradient [18]. Such systems are able to utilise artificial saline solutions, providing opportunities to improve electrochemical potential and hence power density through increasing the concentration gradient, temperature or valence of the salt [19]. Further exergetic advantage can be achieved in closed-loop RED systems by feedwater recycling, which has been demonstrated to increase net energy recovery [20]. This is analogous to ED, where multi-stage or feed recycling configurations are generally proposed to deliver high quality desalinated water [21]. However, feed recirculation introduces complex temporal phenomena due to the cumulative effect of water transport on the concentration gradient, which is exacerbated by the elevated concentration gradient [22]. It is hypothesised that the principles of RED stack design in these scenarios may therefore require closer alignment to those of ED for desalination applications.

The aim of this paper is to determine how to transition from ED stack design towards the practical implementation of an RED stack design suitable for high concentration gradients when operated in recycle, for closed-loop applications. To facilitate engineering rationalisation, commercially available stack designs for ED and RED of equivalent stack dimensions and surface area are initially compared and are benchmarked on concentrated brines in single pass before evaluation in recycle. Subsequent investigation as to the contribution of membrane properties and intermembrane distance on power density and energy efficiency is undertaken during concentrated brine recirculation, in order to better characterise the trade-off between membrane permselectivity and water permeability and to limit exergy losses, respectively.

## 2 Materials and methods

A FumaTech RED-800–2-25 module (FumaTech, Bietigheim-Bissingen, Germany) and MemBrain EDR-Y module (MemBrain, Czech Republic) were tested to determine the performance of commercially available modules using high concentration gradients. Both stacks had dimensions of 10 cm × 40 cm and were equipped with 25 cell pairs, giving a total active membrane area of 2 m^2^ (Table 1)*.* This allowed for a practical comparison between the two stack designs. A custom-made 10 × 10 cm stack was used to identify the contribution of individual stack components on RED performance. By using the same stack there would be no influence of stack features within the endplates in the comparison. Titanium electrodes with Ru/Ir mixed metal oxide coating were fitted into custom-made endplates (Model Products, Bedfordshire, UK). 5 pairs of ion exchange membranes were stacked alternately in the module, separated by nylon woven spacers (SEFAR, Heiden, Switzerland) and silicon gaskets (Silex Silicones Ltd, Hampshire, UK). Membrane type, intermembrane distance and electrode material were individually varied to determine the effect of energy efficiency and work produced from a fixed volume. The membranes tested were homogeneous Neosepta AMX/CMX, Neosepta ACS/CMS (Eurodia Industrie SA, Pertuis, France) and Selemion ASA/CSO (AGC Engineering, Japan), Fumasep FAS-50 and FKS-50 (FumaTech, Bietigheim-Bissingen, Germany) and heterogeneous Ralex AMH-PES and CMH-PES (MemBrain, Czech Republic) (Table 2). These membranes were chosen as they had a broad range of electrochemical (e.g. ion exchange capacity, permselectivity, resistance) and structural characteristics (e.g. thickness). The homogeneous membranes generally exhibit uniformly distributed ionogenic groups throughout the membrane whereas the heterogeneous membranes consist of pockets of ionogenic material distributed in a polymer support matrix. Intermembrane distance was varied by using spacers and gaskets with thicknesses of 0.1 mm, 0.155 mm, 0.2 mm, 0.3 mm and 0.5 mm. Peristaltic pumps recirculated the electrode rinse and dilute and concentrated feeds to the modules (Watson Marlow, Cornwall, UK). Conductivity meters were fitted inline to enable feed concentration to be monitored throughout the experiment (2 CDH-SD1, Omega Engineering Limited, Manchester, UK and 2 Mettler Toledo Seven2Go Pro S7, Wolf Laboratories, York, UK). Feed reservoirs were placed on balances to quantify water flux and enable a full mass balance to be carried out (Kern SFB 20K2HIP, Scales and Balances, Thetford, UK).

**Table 1:** Properties of the two commercially available modules tested.

Module manufacturer	MemBrain	FumaTech
Optimised for	Electrodialysis	Reverse Electrodialysis
Stack size	10 cm × 40 cm	10 cm × 40 cm
Cell pairs	25	25
Total Membrane Area (m^2^)	2	2
Spacer Thickness (mm)	0.8	0.155
Anion Exchange Membrane	Ralex AMH-PES	Fumasep FAS-50
Cation Exchange Membrane	Ralex CMH-PES	Fumasep FKS-50
Membrane Thickness (μm)	714–764	45–55
Electrode material	Titanium MMO	Titanium MMO
Flow Direction	Co-current	Co-current

**Table 2:** Membrane properties from the literature on membranes tested in recycle using a 4 M concentrated feed and 0.02 M dilute feed.

		Membrane type: Anion (+)/Cation (−)	Material/Ionogenic Group*	IEC (mequiv./g dry)	Permselectivity (%)	Resistance (Ω cm^2^)	Thickness (μm)	Membrane Type Homogeneous (Hom) / Heterogeneous (Het)	Ref:
Neosepta	AMX	(+)	PS/DVB, –N(CH_3_)_3_^+^	1.25	90.7	2.35	134	Hom	[11]
	CMX	(−)	PS/DVB, –SO_3_^2−^	1.62	99	2.91	164	Hom	[11]
Neosepta	ACS	(+)	PS/DVB, –N(CH_3_)_3_^+^	1.97 ± 0.01	100 (measured for pair) [13]Monoselective for NaCl	3.8 (data sheet)	117 ± 3	Hom	[30]
	CMS	(−)	Proprietary	2.28 ± 0.05		1.8 (data sheet)	136 ± 3	Hom	[30]
Ralex	AMH-PES	(+)	–N(CH_3_)_3_^+^	1.97	89.3	7.66	714	Het	[11]
	CMH-PES	(−)	-SO_3_^2−^	2.34	99	11.33	764	Het	[11]
Fumasep	FAS-50	(+)	Proprietary	1.6–2.0	92–96	0.6–1.5	45–55	Hom	Data sheet
	FKS-50	(−)	–SO_3_^2−^	1.2–1.4	97–99	1.8–2.5	45–55	Hom	Data sheet
Selemion	ASA	(+)	Proprietary	2.13 ± 0.04	–		96 ± 3	Hom	[30]
	CSO	(−)	PS/DVB, –SO_3_^2−^	2.2 ± 0.02	95	1.91	97 ± 2	Hom	[30]

*PS/DVB = styrene – divinylbenzene copolymer.

### 2.1 Preparation of solutions

Sodium chloride (NaCl) solutions were prepared for the concentrated and dilute feeds using 99% NaCl (Alfa-Aesar, Lancashire, UK) and deionised water. For the concentrated feed, solutions of 0.5 M, typically used to represent sea water in the literature, and 4 M were prepared. For the dilute feed, a 0.02 M solution was prepared. The electrode rinse consisted of 0.1 M potassium ferricyanide (K_3_Fe(CN)_6_), 0.1 M potassium ferrocyanide (K_4_Fe(CN)_6_)(Fisher Scientific, Leicestershire, UK) and 2 M or 0.25 M NaCl (Alfa-Aesar, Lancashire, UK) depending on the concentration of the feed to limit water transport to the electrolyte and was continuously recirculated to the stacks. For experiments using the large commercially available stacks, a feed reservoir containing 5 L of electrode rinse solution was prepared and 1 L for the 10 cm × 10 cm stack and wrapped in tin foil to avoid exposure to light.

### 2.2 Electrochemical measurements

A potentiostat (IviumStat.h, Alvatek, UK) was used to make electrochemical measurements, with the data logged using IviumSoft (IviumStat.h, Alvatek, UK). A consistent open circuit voltage < 0.01 V s^−1^ was obtained before electrochemical measurements were made to ensure steady-state. All measurements were carried out at least three times, with the mean reported and error bars used to represent the standard deviation of the triplicate. In single pass, chronopotentiometry was carried out at a range of current densities until a stable voltage was achieved. Power density was calculated:

(1) $$P_{d}=\frac{\mathrm{UI}}{\;A}$$

where I is the current (A), U is the voltage (V) produced by the stack and A is the total active membrane area (m^2^).

To determine energy efficiency, feeds were recirculated with the potentiostat set at a constant current, enabling total work produced ($W_{\mathrm{RED}}$) to be calculated:

(2) $$W_{\mathrm{RED}}=\sum_{t_{o}}^{t_{\mathrm{end}}}UI\Delta t$$

Where t_o_ is the time at which work was produced and t_end_ the time stopped, U is the voltage produced (V) and I is the current [18]. The Gibbs free energy in the system ($\Delta G_{\mathrm{mix}}$) is the theoretical maximum energy which can be recovered, assuming total mixing and no exergy losses:

(3) $$\Delta G_{\mathrm{mix}}=\Delta G_{m}-\left(\Delta G_{c}+\Delta G_{d}\right)$$

where ΔG_mix_ is the Gibbs energy (J) and the subscripts m, c and d represent the mixed outlet stream, concentrated and dilute feeds respectively. For ideal solutions:

(4) $$\Delta G_{\mathrm{mix}}=-\left(N_{c}+N_{d}\right)T\Delta S_{m}-\left(-N_{c}T\Delta S_{c}-N_{d}T\Delta S_{d}\right)$$

where N is the number of moles (mol), T is the temperature (K) and ΔS is the molar entropy (J K^−1^ mol^–1^). The molar entropy is determined by:

(5) $$\Delta S=-R\sum_{i}x_{i}\ln x_{i}$$

where R is the universal gas constant and x is the mole fraction of species i. [6]. The energy efficiency $\left(\eta_{\mathrm{RED}}\right)$ is defined as the ratio of work produced to the total available Gibbs free energy:

(6) $$\eta_{\mathrm{RED}}=\frac{W_{\mathrm{RED}}}{\Delta G_{\mathrm{mix}}}x100$$

## 3 Results and discussion

**Fig. 1:**
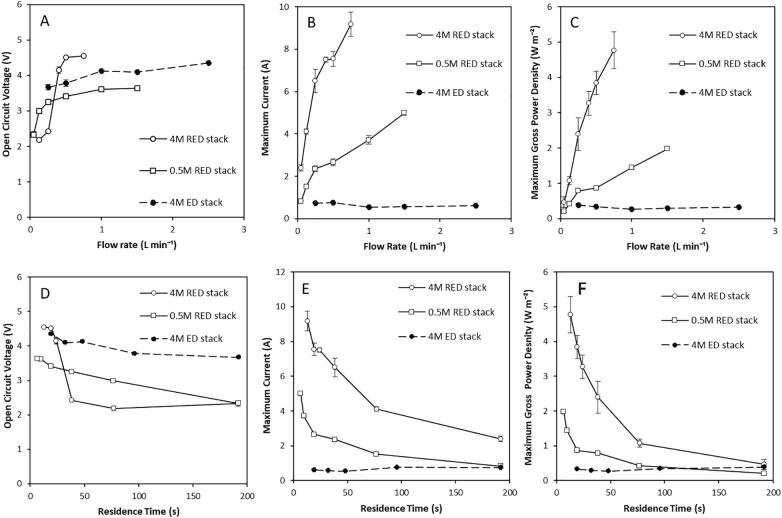
(A) Open circuit voltage; (B) maximum current; and (C) maximum gross power density obtained by commercially available stacks optimized for RED and ED at range of flow rates; and (D) open circuit voltage (E) maximum current and (F) maximum power density against feed residence time. Dilute feed of 0.02 M and a concentrated feed of 0.5 M and 4 M in single pass. Error bars represent the standard deviation of a triplicate.

The performance of commercially available stacks for high concentration reverse electrodialysis was experimentally determined. Measurements were initially carried out in single pass to evidence the maximum power density that can be achieved using the two commercially available modules when operated at a large concentration difference. The two units had an equal total active membrane area and stack dimensions, but different ion exchange membranes and intermembrane distances (Table 1). For both stacks, the open circuit voltage (OCV) improved as flow rate was increased up to a plateau at approximately 4.5 V, however this occurred at a lower flow rate of 0.5 L min-1 in the RED stack, compared to 1 L min-1 in the ED stack (Fig. 1A). This OCV was significantly lower than the theoretically calculated OCV of 6.6 V, which is attributed to the uncontrolled migration of water and ions across the membranes [23], driven by the elevated concentration gradient. For the RED stack, current increased with flow rate up to a maximum of 9.2 A at 0.75 L min^−1^. This was explained by the increase in the rate of ionic transfer, facilitated by the higher flow rate, which reduced concentration polarisation [24]. By contrast, current was limited to approximately 0.6 A in the ED stack and remained unchanged as flow rate was increased up to a flow rate of 2.5 L min^−1^ (Fig. 1B). This cannot be attributed to ion exchange capacity as the FAS-50/FKS-50 membranes in the RED module and the AMH-PES/CMH-PES membranes in the ED module had a similar ion exchange capacity (Table 2). However, membrane thickness varied significantly between the RED and ED modules, which were 0.05 and 0.7 mm thickness respectively. Membrane thickness is directly correlated with resistance [25], and heterogeneous membranes with non-uniform charge distribution [26] such as those in the ED stack have much higher resistance than homogenous ones, as in the RED stack [11]. For seawater and river water feeds, low resistance membranes are prioritised to improve power density by RED [12]. However, for ED, low water permeability membranes are preferred to reduce energy requirements for desalination [6]. A maximum gross power density of 0.33 W m^−2^ was produced by the ED stack in comparison to 4.78 W m^−2^ for the RED stack (Fig. 1C). This difference is attributed to intrinsic membrane resistance [25], however, the significant difference in intermembrane distance may also play a role.

As stack dimensions and cell pair number in the two stacks were equal, the velocity at a given flow rate is determined by the intermembrane distance. The intermembrane distance of the ED stack was over four times greater than that in the RED stack, recorded as 0.8 mm and 0.15 mm respectively, and therefore residence time (τ) was similarly varied for a given flow rate $\left(\tau =v/l\right)$. Residence time has previously been used to estimate performance across process scales, with a deterioration in power density observed for extended residence times [23]. To account for this, the performance of the two stacks was also evaluated in terms of residence time. For residence times greater than 30 s, the ED stack produced a higher OCV than the RED stack at an equivalent residence time (Fig. 1D). This is likely to be because the increased thickness of the ED membranes mitigates exergy losses by retarding water and ionic flux. However, current was significantly lower in the ED stack (Fig. 1E). Increased intermembrane distance is expected to increase internal ohmic resistance [8], with Długołecki et al. [11] reporting that for an intermembrane distance of >0.6 mm, spacer resistance dominates, and membrane properties have very little effect on RED performance for feeds with an equivalent concentration to seawater and river water. Therefore, in this study, the increased spacer thickness which is advantageous to ED, maybe similarly limiting for RED with concentrated brines. Due to the reduction in current, gross power densities were significantly lower in the ED stack compared with the RED stack (Fig. 1F). To illustrate, at a constant residence time of 20 s, a maximum current of 0.06 A was obtained compared to 7.5 A, producing gross maximum power densities of 0.33 W m^−2^ and 3.85 W m^−2^ respectively.

**Fig. 2:**
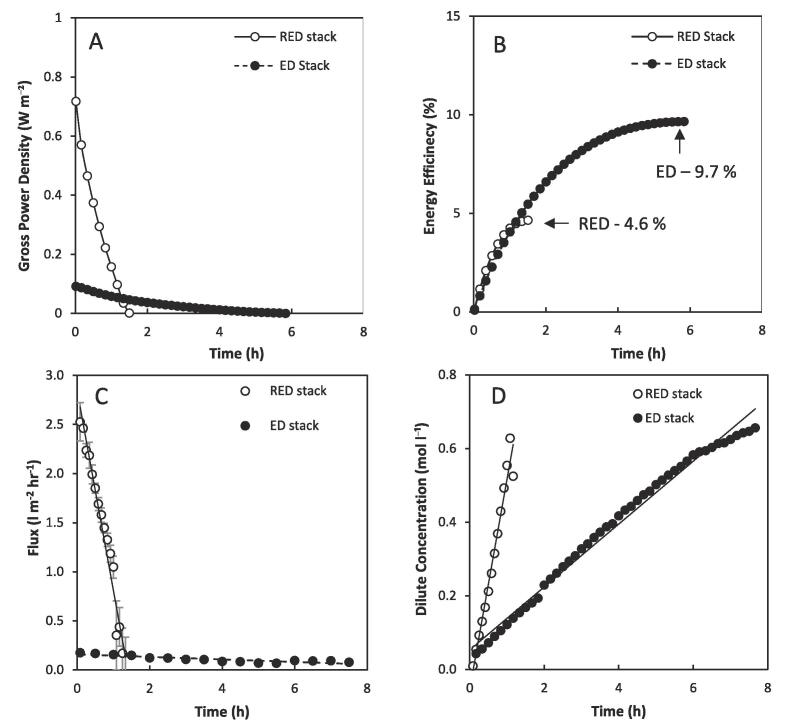
(A) Power density; (B) energy efficiency; (C) water flux; and (D) dilute concentration over time obtained using commercially available stacks optimized for RED and ED in recycle. Dilute feed, 5 kg 0.02 M NaCl; concentrated feed 5 kg 4 M NaCl. Flow rate, 0.5 L min^−1^; current, 400 mA. Error bars represent the standard deviation of a triplicate.

The ten-fold increase in power density using the RED stack demonstrates that this ED stack design is not suitable for delivering high power density from concentrated brines in single pass. However, the distinction in ED stack design including increased intermembrane distance that promotes longer residence times, and increased membrane thickness, could benefit total energy recovery during recycle, due to a reduction in water transport. Consequently, the two stacks were compared in recycle by fixing a high concentration gradient (4 M and 0.02 M feeds) at a current of 0.4 A (current density, 10 A m^−2^), which is the highest current that could be sustained by the ED stack. At an equal flow rate of 0.5 L min^−1^ a maximum gross power density of ~ 0.1 W m^−2^ was obtained in the ED stack, compared to 0.72 W m^−2^ in the RED stack (Fig. 2A). Although maximum power densities obtained using the ED module in single pass and recycle operation are similar, maximum power output achieved by the RED module is significantly reduced. This is because the power density is limited by the reduced current utilised in recycle to enable comparison between modules. The difference in power density obtained by the two stacks at equivalent current and flow rate can be partly explained by the difference in residence time of 96 s and 19 s, in the ED and RED stack, respectively. A total energy efficiency of 9.7%, equivalent to 0.63 kJ kg^−1^ was achieved using the ED stack at these conditions (Fig. 2B). However, half the energy efficiency, 4.6%, was obtained using the RED stack. This difference is attributed to the increased water flux in the RED stack (Fig. 2C). This introduced a sharp decline in the concentration gradient and hence electrochemical potential, as illustrated by the increased saline concentration of the dilute feed over a shorter time interval (Fig. 2D). Significant decreases in energy efficiency due to water transport have been previously reported when using large concentration gradients [16]. Membranes with low water permeability are favoured for ED as they decrease exergy loss due to water transport [6], and this demonstrates that membranes with low water permeability could also improve efficiency in RED using concentrated brines and in recycle applications.

### 3.1 Effect of membrane properties on high concentration reverse electrodialysis

**Fig. 3:**
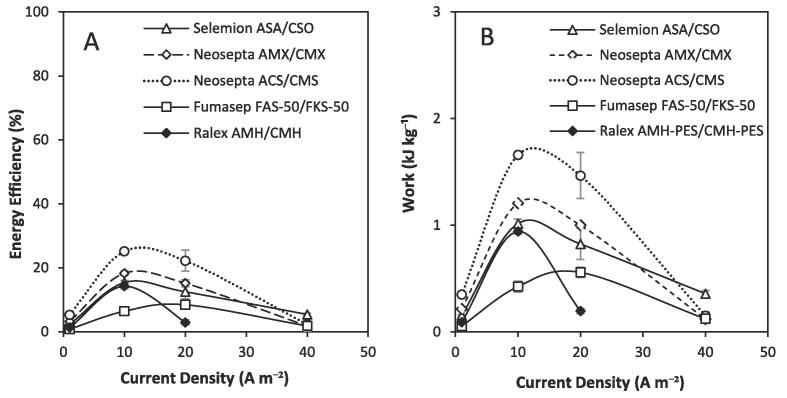
(A) Energy efficiency obtained and (B) work produced using commercially available membranes in recycle at a range of current densities using a 10 cm × 10 cm 5-cell pair RED stack and an intermembrane distance of 0.3 mm. Dilute feed, 0.25 kg 0.02 M; concentrated feed, 0.25 kg 4 M; flow rate, 0.2 L min^−1^. Error bars represent the standard deviation of a triplicate.

The effect of membrane properties on power density and energy efficiency using concentrated brines in recycle were discretely investigated, using five pairs of commercially available IEMs (Table 2), which included those used in the commercially available RED and ED stacks (Fumasep FAS-50/FKS-50 and Ralex AMH-PES/CMH-PES respectively). Although these membranes generally contained the same ionogenic groups, their structural and morphological properties differed (Table 2). Current density was varied to identify an optimum energy efficiency for each IEM cell pair (Fig. 3A). The increase in energy efficiency with current density is expected in RED, as in contrast to ED, water transport is reduced as the corresponding increase in electro-osmosis counteracts the disadvantage of osmotic flux [9]. However, above an optimal current density, high internal resistances inhibit performance [10]. A peak in energy efficiency was therefore observed at 10 A m^−2^ for the Selemion ASA/CSO, Neosepta AMX/CMX, Neosepta ACS/CMS and Ralex AMH-PES/CMH-PES membranes, whereas a peak occurred at a higher current density of 20 A m^−2^ for the Fumasep FAS-50/FKS-50 membranes. This implies that higher power densities can be promoted through the Fumasep FAS-50/FKS-50 IEMs, which may explain their frequent selection for seawater/ river water applications. For closed-loop RED applications, the energy efficiency (specifically, the work produced per kg of feed (Fig. 3B)) is a critical determining factor in overall system efficiency [28]. Despite the increased current, the energy efficiency obtained using the Fumasep membranes was significantly lower than all other membranes pairs, at 8.5% (Fig. 3A). The Ralex AMH-PES/CMH-PES membranes from the ED stack obtained an improved efficiency of 14.2%, illustrating that they can benefit the performance of RED using concentrated brines. This improvement can be attributed to a reduction in osmosis of the Ralex membranes. This likely arises in part from the much larger thickness of these membranes which will decrease water permeance [26]. The heterogeneous structure of these membranes may also be contributing to their low water transport compared to the homogeneous membranes as the structural morphology of ion exchange membranes is known to also influence their water transport properties [29].

**Fig. 4:**
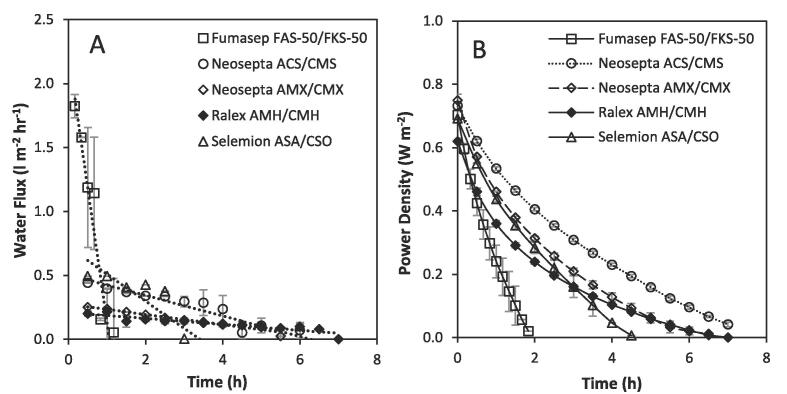
(A) Water flux and (B) gross power density over time using commercially available membranes in recycle in a 10 cm × 10 cm 5-cell pair RED stack with an intermembrane distance of 0.3 mm. Dilute feed, 0.25 kg 0.02 M; concentrated feed, 0.25 kg 4 M; flow rate, 0.2 L min^−1^, current density 10 A m^−2^. Error bars represent the standard deviation of a triplicate.

For each IEM cell pair, water flux was observed to decrease over time as the concentration gradient and hence the driving force for water transport decreased (Fig. 4A). A water flux of 1.82 L m^−2^h^−1^ was recorded across the Fumasep FAS-50 membranes, which was the highest across the 5 IEM cell pairs tested and was an order of magnitude greater than that of the Ralex (AMH/CMH) and Neosepta (AMX/CMX) membranes, which exhibited the lowest water flux. The greater energy efficiency of the Neosepta (AMX/CMX) membranes over the the Ralex (AMH/CMH) at this similar water flux but vastly different thicknesses and resistances indicates though that a homogeneous bulk structure ion exchange membrane is preferred for high concentration RED in recycle. For this set of conditions, the maximum gross power density recorded from the Fumasep FAS-50/FKS-50 IEMs at a current density of 10 A m^−2^ was 0.7 W m^−2^. This was comparable to both Neosepta IEMs that were characterised by comparable ohmic resistances (Table 2) and was quite similar to power densities obtained from the Ralex AMH-PES/CMH-PES membranes used within the ED stack, which confer considerably higher membrane resistances between 7.6 and 11.3 Ω cm^−2^, characteristic of heterogeneous ion exchange membranes [30] (Table 2). The relative insensitivity of power density to membrane resistance can be attributed to the OCV that can be sustained for Ralex IEMs, which enables comparatively higher local concentration gradients for a longer period in comparison to the thinner membranes. The increased rate of water transfer is detrimental to energy recovery, due to the faster deterioration in salinity gradient, which reduces temporal power production as the electrochemical potential tends toward 0 V (Fig. 4B). Membranes comprising lower water permeability can reduce such exergy losses and improve energy efficiency, as theoretically demonstrated by Giacalone et al. [31]. Water permeability can be controlled through modification of membrane microstructure to reduce water permeance, or by increasing the membrane thickness [32]. In this study, increasing membrane thickness is demonstrated to improve energy efficiency for RED in recycle, however the corresponding increase in areal resistance reduces obtainable power densities [26] leading to a critical trade-off. In river water/sea water applications, membrane resistance has therefore been perceived as the primary membrane property governing power density [26]. Whilst results from this study broadly agree with this assertion in single pass, the highest power density of 0.73 W m^−2^ and energy efficiency of 25% in recycle was obtained using the homogeneous Neosepta ACS/CMS membranes, which comprise of an intermediate thickness and similar or greater resistance than competing IEMs. The apparent improvements in power density and energy efficiency can be attributed to the reduction in water transport, emphasising that a more holistic approach is required to membrane selection when subjected to high concentration gradients in recycle.

### 3.2 Effect of intermembrane distance on high concentration reverse electrodialysis

**Fig. 5:**
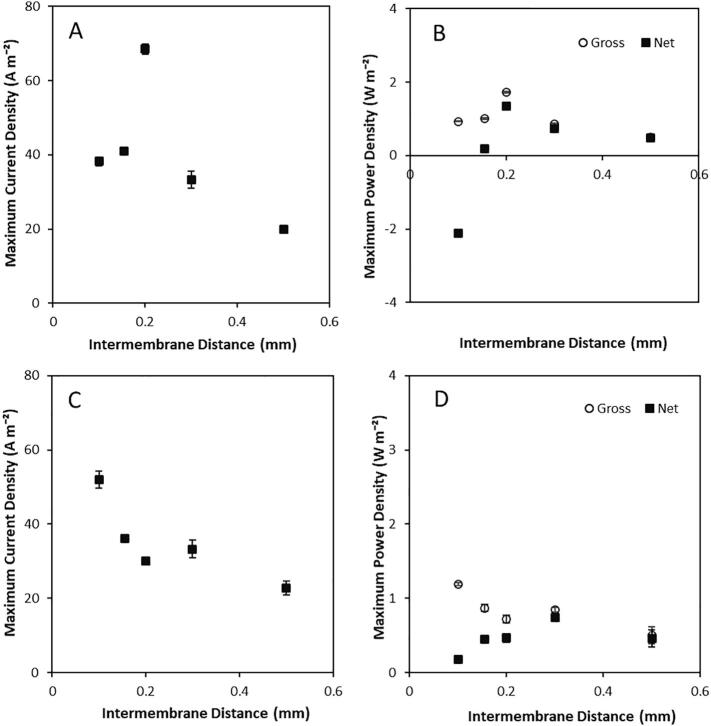
Spacer thickness in single pass on (A) maximum current and (B) maximum power density at a constant flow rate of 0.2 L min^−1^ and (C) maximum current and (D) maximum power density at constant velocity of 0.22 cm s^−1^ using a 10 cm × 10 cm RED stack containing 5 pairs Neosepta AMX/CMX membranes. Dilute feed, 0.25 kg 0.02 M; concentrated feed, 0.25 kg 4 M; current, 100 mA. Error bars represent the standard deviation of a triplicate.

The intermembrane distance was initially varied in single pass, to determine whether reduced intermembrane distances improve the gross and net power density obtained by RED using a large concentration gradient. At a constant flow rate of 0.2 L min^−1^, current increased to an optimum of 68 A m^−2^ as intermembrane distance was increased to 0.2 mm, before rapidly decreasing as intermembrane distance was increased further (Fig. 5A). The peak current recorded at 0.2 mm was coincident with a maximum gross power density of 1.72 W m^−2^ (Fig. 5B). The reduction in power density above this intermembrane distance can be ascribed to ohmic and boundary layer resistances which are thought to increase as intermembrane distances increases, subsequently reducing current [8]. Net power density accounts for the hydraulic pumping losses. For small intermembrane distances of 0.1 mm, net power was negative, indicating that more pumping power was required than was produced by RED. Net power density increased up to 1.34 W m^−2^ at an intermembrane distance of 0.2 mm due to the increased gross power density. Vermaas et al. [8] reported decreased net power below an optimal intermembrane distance of 0.1 mm using artificial seawater/river water. A thinner intermembrane distance is required for lower concentration gradient (sea/ river) feeds due to the reduced gross power densities obtained, compared to the utilisation of higher concentration gradients in this study, which vastly improved gross power density, enabling use of increased intermembrane distances.

The transition in intermembrane distance at a fixed flow rate results in different velocities and residence times. Consequently, experiments were undertaken at a constant velocity of 0.22 cm s^−1^, equating to a residence time of 4.5 s for each intermembrane distance studied (Fig. 5C). Current decreased approximately linearly as the intermembrane distance was increased (Fig. 5C) due to ohmic and boundary layer resistance [8]. Below 0.3 mm, gross power densities are lower than the values obtained at 0.2 L min^−1^ (Fig. 5D). Whilst this is an artefact of the lower flow rate required to obtain a comparable velocity, RED will be scaled up based on velocity and residence time to ensure performance is consistent between process scales to account for concentration polarisation and spacer shadow effects [27]. Consequently, the intermembrane distance that provides the highest net power density at comparable velocity can be assumed to represent the most favourable condition. Whilst the highest gross power density of 1.18 W m^−2^, corresponding to a net power density of 0.17 W m^−2^, was obtained at a 0.1 mm intermembrane distance, the maximum net power density of 0.74 W m^−2^ was recorded with a 0.3 mm intermembrane distance (Fig. 5D). This is at the upper limit of those ordinarily rationalised for seawater/river water RED, and therefore confirms the assertion that slightly wider intermembrane distances may be appropriate for power production from high concentration brines.

**Fig. 6:**
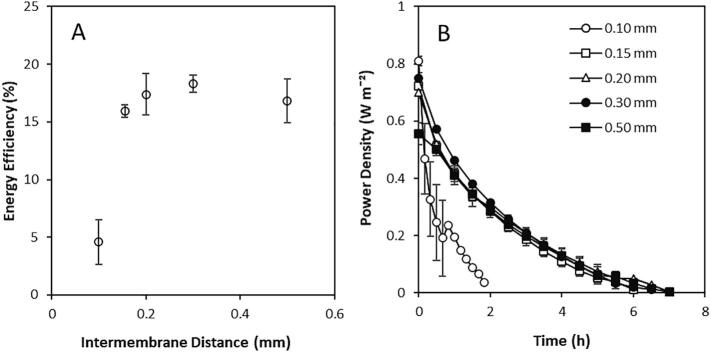
Effect of spacer thickness on (A) energy efficiency and (B) power density over time using 4 M and 0.02 M feeds in recycle in a 10 cm × 10 cm RED stack containing 5 pairs Neosepta AMX/CMX membranes at a constant velocity of 0.22 cms^−1^, current applied 100 mA. Error bars represent the standard deviation of a triplicate.

The role of intermembrane distance on energy efficiency was subsequently studied for the recycling of high concentration gradient brines (Fig. 6A) Intermembrane distances were compared at a fixed velocity of 0.22 cm s^−1^, corresponding to a residence time of 4.5 s. An energy efficiency of just 4.5% was obtained for an intermembrane distance of 0.1 mm (Fig. 6A). This was attributed to the energy demanded by the higher pressure drop for very thin intermembrane distances [8]. A rapid decline in power density was also observed at 0.1 mm intermembrane distance (Fig. 6B), which was attributed to the reduced stack volume which constrained power production. However, a plateau in energy efficiency of around 16% was established for intermembrane distances exceeding 0.3 mm, coupled with comparable power densities. This indicates that, similar to ED applications, larger spacer thickness improves RED energy recovery for high concentration gradients in recycle.

## 4 Conclusions

In this study, stack features conventionally associated with ED are demonstrated to improve energy recovery of RED utilising concentrated brines in recycle. Improved power density of 4.78 W m^−2^ was obtained using an RED module compared to 0.33 W m^−2^ using an ED module, demonstrating its suitability for high salinity gradient applications. However, energy efficiency doubled from 4.6% in the RED stack to 9.7% using the ED stack when operated at low current densities (0.1 A) in recycle. This increase in efficiency was attributed to the longer residence time at a fixed flow rate due to the larger intermembrane distance of 0.80 mm compared to 0.16 mm as well as reduced exergy losses from water transport facilitated by the membrane properties. Investigation of ion exchange properties demonstrated thehighest power density and energy efficiency of 0.73 W m^−2^ and 25% respectively from Neosepta ACS/CMS membranes, due to their reduction in water permeability and resistance complemented with an increased membrane permselectivity. In contrast to sea/river applications where thinner intermembrane distances of approximately 0.1 mm are typically preferred, increasing the intermembrane distance to 0.3 mm improved net power density from 0.17 W m^−2^ to 0.74 W m^−2^, and energy efficiency from 4.5% to 16%. Evidence from this study, demonstrates that adopting intermembrane distances and membrane properties traditionally more aligned with ED stack design, can benefit RED stack performance performance when utilising concentrated brines. The system level improvements identified in this research increase viability of RED as a next generation technology for energy storage and thermal to electrical conversion, which are critical to the net zero transition. In addition to identifying improvements to energy efficiency, diagnosis of power density emphasised the critical role of membrane properties for closed-loop systems characterised by elevated Gibbs free energy, which will inevitably contribute to the levelised cost of energy for RED, and should therefore be to guide future research direction.

## Declaration of Competing Interest

The authors declare that they have no known competing financial interests or personal relationships that could have appeared to influence the work reported in this paper.
